# A polyphenolic cinnamon fraction exhibits anti-inflammatory properties in a monocyte/macrophage model

**DOI:** 10.1371/journal.pone.0244805

**Published:** 2021-01-13

**Authors:** Amel Ben Lagha, Jabrane Azelmat, Katy Vaillancourt, Daniel Grenier

**Affiliations:** Oral Ecology Research Group, Faculty of Dentistry, Université Laval, Quebec City, QC, Canada; University of the Pacific, UNITED STATES

## Abstract

Periodontal diseases are bacteria-induced inflammatory disorders that lead to the destruction of the tooth-supporting tissues. Active compounds endowed with a capacity to regulate the inflammatory response are regarded as potential therapeutic agents for the treatment of periodontal diseases. The aim of this study was to characterize the anti-inflammatory properties of a polyphenolic cinnamon fraction. Chromatographic and mass spectrometry analyses of the polyphenolic composition of the cinnamon fraction revealed that phenolic acids, flavonoids (flavonols, anthocyanins, flavan-3-ols), and procyanidins make up 9.22%, 0.72%, and 10.63% of the cinnamon fraction, respectively. We used a macrophage model stimulated with lipopolysaccharides (LPS) from either *Aggregatibacter actinomycetemcomitans* or *Escherichia coli* to show that the cinnamon fraction dose-dependently reduced IL-6, IL-8, and TNF-α secretion. Evidence was brought that this inhibition of cytokine secretion may result from the ability of the fraction to prevent LPS-induced NF-κB activation. We also showed that the cinnamon fraction reduces LPS binding to monocytes, which may contribute to its anti-inflammatory properties. Lastly, using a competitor assay, it was found that the cinnamon fraction may represent a natural PPAR-γ ligand. Within the limitations of this *in vitro* study, the cinnamon fraction was shown to exhibit a therapeutic potential for the treatment of periodontal diseases due to its anti-inflammatory properties.

## Introduction

Inflammation plays an important role in the host immune defense response to harmful stimuli such as damaged cells, irritants, and pathogens. This complex process not only eliminates the primary cause of infection or tissue injury, it also eradicates apoptotic/necrotic cells and damaged tissue and initiates tissue repair. Immune cells, including macrophages, neutrophils, and lymphocytes, respond to infectious agents by modulating an inflammatory response [1]. The nuclear factor kappa B (NF-κB) pathway plays an essential role in the inflammatory response by promoting the expression of a broad array of inflammatory mediators [2, 3]. A dysregulation of the secretion of inflammatory mediators by immune cells can lead to a variety of chronic inflammatory disorders, including periodontal diseases.

Periodontal diseases are a group of multifactorial inflammatory disorders that lead to the destruction of the supporting structures of the teeth. They are induced and modulated by a complex dysbiotic microbiota, mostly Gram-negative anaerobic bacteria that colonize the subgingival areas [4, 5]. The host inflammatory response to periodontal pathogens modulates the progression and severity of periodontitis, a form of periodontal disease that causes irreversible damage to connective tissues and alveolar bone [5, 6]. Following a challenge of the innate defense system with periodontal bacteria, a complete resolution of the acute inflammatory response is necessary to return to homeostasis and periodontal health. However, an excessive host inflammatory response to a continuous challenge by a pathogenic periodontal biofilm and/or an inadequate resolution of inflammation can initiate chronic periodontitis. In the last two decades, a lot of research has been carried out to identify modifiable (poor oral hygiene, tobacco smoking, diabetes mellitus, etc) and non-modifiable (age, genetic factors, etc) risk factors that can impact both the oral microbial composition and the progression of periodontitis [7, 8].

Macrophages, which originate either from circulating monocytes or from embryo-derived precursors, are found in higher numbers in active periodontal lesions than in inactive sites [9]. The continuous excessive secretion of various cytokines and matrix metalloproteinases (MMPs) by macrophages following the recognition of periodontal bacteria by the toll-like receptor pathway modulates periodontal tissue destruction [1, 10, 11]. Active compounds endowed with a capacity to down-regulate the inflammatory response of macrophages may thus be regarded as potential new therapeutic agents for the treatment of periodontal diseases [11, 12].

Cinnamon (*Cinnamomun* spp., Lauraceae family) is a widely used culinary herb mainly grown in Asia, China, and Australia. Cinnamon essential oils and extracts have been reported to possess various therapeutic properties for a number of human diseases and disorders [13, 14]. More specifically, essential oils isolated from different parts of cinnamon (leaves, bark, flowers) have been reported to exert marked antimicrobial effects against different oral pathogens associated with dental caries, periodontal diseases, and endodontic infections [15]. In a recent study, we provided evidence that a polyphenolic cinnamon fraction may have a beneficial effect on oral health by reducing the ability of *Candida albicans*, a pathogenic fungus that causes oral candidiasis, to form biofilm and adhere to oral epithelial cells [16]. In addition, this fraction reinforced the epithelial barrier function in a double chamber *in vitro* model, as shown by an increase of transepithelial electrical resistance, which is a widely used method to assess the integrity of tight junction dynamics. The aim of the present study was to further characterize the polyphenolic cinnamon fraction with respect to its anti-inflammatory properties using a monocyte/macrophage model.

## Materials and methods

### Cinnamon extract and polyphenolic characterization

A cinnamon bark aqueous extract (*Cinnamomum burmanii*) commercialized as Cinnulin PF^®^ was kindly provided by IN Ingredients Inc. (Spring Hill, TN, USA). A stock solution (10 mg/mL) was prepared in distilled water, filtered through a 0.2-μm pore-size membrane filter, and kept at 4°C in the dark. Anthocyanins and procyanidins were characterized by high-performance liquid chromatography (HPLC) using delphinidin 3-glucoside and epicatechin as standards, respectively [17]. Phenolic acids and flavonoids were characterized as previously described by Dudonne et al. [17] using an Acquity^®^ ultra-performance liquid chromatography-tandem mass spectrometer (UHPLC-MS/MS) coupled to a triple quadrupole (TQD) mass spectrometer equipped with a Z-spray electrospray interface (Waters Ltd., Mississauga, ON, Canada). Commercial phenolic and flavonoid standards were analyzed using the same parameters and were used for quantification purposes.

### Treatment of macrophages

The monoblastic leukemia cell line U937 (ATCC CRL-1593.2) was cultivated in RPMI-1640 medium (HyClone Laboratories, Logan, UT, USA) supplemented with 10% heat-inactivated fetal bovine serum (FBS) (RPMI-FBS) and 100 μg/mL of penicillin-streptomycin and was incubated at 37°C in a 5% CO_2_ atmosphere. The protocol described by Rovera et al. [18] was used to induce the differentiation of the monocytes into adherent macrophages using phorbol myristic acid. Adherent macrophages were harvested by scraping, washed twice by centrifugation (200 x *g* for 8 min), suspended in RPMI with 1% heat-inactivated FBS at a density of 1 x 10^6^ cells/mL, and seeded in a 6-well plate (2 x 10^6^ cells/well in 2 mL). After an overnight incubation at 37°C in a 5% CO_2_ atmosphere, the macrophages were treated (2 h) with increasing concentrations (32.5 to 500 μg/mL; in RPMI containing 1% FBS) of the cinnamon fraction prior to stimulating them with LPS from either *Aggregatibacter actinomycetemcomitans* or *Escherichia coli* at a final concentration of 1 μg/mL. *A*. *actinomycetemcomitans* ATCC 29522 LPS was prepared using the protocol described by Darveau and Hancock [19] while *E*. *coli* LPS was purchased from Sigma-Aldrich Canada (Oakville, ON, Canada). After a 24-h incubation (37°C in 5% CO_2_), the culture medium supernatants were collected and were stored at –20°C until used to assay the cytokines. Cells incubated in culture medium with or without the cinnamon fraction, but not stimulated with LPS, were used as controls.

### Determination of cytokine production

Commercial enzyme-linked immunosorbent assay (ELISA) kits (R&D Systems, Minneapolis, MN, USA) were used to quantify IL-6, IL-8, and TNF-α concentrations in the cell-free culture supernatants according to the manufacturer’s protocols. The absorbance at 450 nm was read in a Synergy 2 microplate reader (Bio-Tek Instruments, Winooski, VT, USA) with the wavelength correction set at 550 nm.

### LPS-induced NF-κB activation

The human monoblastic leukemia cell line U937 3xκB-LUC was used to investigate the effect of the cinnamon fraction on LPS-induced NF-κB activation. This subclone of the U937 cell line, which is stably transfected with a luciferase gene coupled to a promoter with three NF-κB-binding sites [20], was kindly provided by R. Blomhoff (University of Oslo, Norway). Cells were cultivated in RPMI-1640 supplemented with 10% heat-inactivated FBS, 100 μg/mL of penicillin G/streptomycin, and 75 μg/mL of hygromycin B at 37°C in a 5% CO_2_ atmosphere. Cells were seeded in the wells (10^6^ cells) of a black wall, black bottom 96-well microplate (Greiner Bio-One North America Inc., Monroe, NC, USA), pre-incubated with increasing concentrations of the cinnamon fraction (32.5 to 500 μg/mL in RPMI containing 1% FBS) for 30 min, and then stimulated for 6 h with LPS from either *A*. *actinomycetemcomitans* or *E*. *coli* at a final concentration of 1 μg/mL. Cells incubated in culture medium without LPS or the cinnamon fraction were used as controls. An assay using the commercial NF-κB inhibitor BAY-11-7082 (5 μg/mL; EMD Millipore Canada, Mississauga, ON, Canada) was used as a positive control for the inhibition of the NF-κB signaling pathway. NF-κB activation was determined by monitoring luciferase activity following the addition of Bright-Glo reagent (Promega Corporation, Madison, WI, USA) in accordance with the manufacturer’s protocol. Luminescence was measured using a Synergy 2 microplate reader.

### Binding of LPS to U937 cells

U937 cells were cultivated as described above and were suspended at a concentration of 10^7^ cells/mL in 50 mM phosphate-buffered saline (PBS; pH 7.2) containing 1% bovine serum albumin (BSA). The cinnamon fraction (final concentrations of 250 and 500 μg/mL) was added (5 μL) to 100 μL of the cell suspension, which was then gently mixed for 30 min at 37°C in a 5% CO_2_ atmosphere. *E*. *coli* LPS conjugated to Alexa 488 (5 μg/ml; Molecular Probes, Eugene, OR, USA) was added (1 μL), and the mixture was incubated for a further 30 min prior to adding 1 mL of PBS-1% BSA. The binding of LPS-Alexa 488 to U937 cells was measured by flow cytometry as previously described [21]. Briefly, Alexa 488 was excited using a 488 argon-ion laser line and was monitored on channel FL1 using a 530-nm emission filter (Beckman Coulter Inc., Indianapolis, IN, USA). The mean fluorescence of 10,000 cells was determined. The LPS binding assay was performed in quadruplicate, and a representative set of data is presented.

### PPAR-γ binding assay

The Green PolarScreen PPARγ Competitor Assay (ThermoFisher Scientific, Waltham, MA, USA), which is based on the reactivity of the binding domain of recombinant human PPAR-γ (peroxisome proliferator-activated receptor gamma) with a specific fluorescent PPAR-γ ligand, was used according to the manufacturer’s protocol. The PPAR-γ/PPAR-γ ligand complex emits fluorescence, which is reduced in the presence of an unlabeled competitor. The cinnamon fraction (final concentrations of 15, 30, and 60 μg/mL) was tested as a potential competitor. Rosiglitazone (40 μM; Thermo Fisher Scientific) was used as a positive unlabeled competitor control. Fluorescence was measured using a Synergy 2 microplate reader with the excitation and emission wavelengths set at 485 and 528 nm, respectively.

### Statistical analysis

Unless indicated otherwise, all assays were performed in triplicate in two independent experiments. The data are expressed as means ± standard deviations (SD). Statistical analyses were performed using a one-way and two-way ANOVA analysis of variance with a post hoc Bonferroni multiple comparison test (GraphPad Software Inc., San Diego, CA, USA). Results were considered statistically significant at p < 0.01.

## Results

The polyphenolic composition of the cinnamon fraction, as determined by chromatographic and MS analyses, is reported in Table 1. Phenolic acids, flavonoids (flavonols, anthocyanins, flavan-3-ols), and procyanidins made up 9.22%, 0.72%, and 10.63% of the cinnamon fraction, respectively. Cinnamic acid was the most abundant phenolic acid (78.63%). The procyanidin content was mostly trimers and tetramers.

**Table 1:** Polyphenolic composition of the cinnamon fraction.

	Composition	Amount (mg/100 g dry weight)
**PHENOLIC ACIDS**		**9 223**
	Caffeic acid	ND
	Chlorogenic acid	21
	Cinnamic acid	7 252
	*o*-Coumaric acid	291
	*p*-Coumaric acid	224
	Cryptochlorogenic acid	ND
	Ferulic acid	48
	Gallic acid	ND
	*p*-Hydroxybenzoic acid	269
	Isoferulic acid	ND
	Neochlorogenic	ND
	Protocatechuic acid	417
	Salicylic acid	701
	Sinapic acid	ND
**FLAVONOIDS**		**722**
**Flavonols**		**296**
	Isorhamnetin	ND
	Kaempferol	ND
	Kaempferol glucoside	11
	Myricetin	ND
	Myricetin glucoside	ND
	Phlorizin	169
	Quercetin	21
	Quercetin galactoside	ND
	Quercetin glucoside	13
	Quercetin rhamnoside	82
	Quercetin rutinoside	ND
**Anthocyanins**		**ND**
**Flavan-3-ols**		**426**
	Catechin	273
	Epicatechin	153
**PROCYANIDINS**		**10 630**
	Monomers	459
	Dimers	935
	Trimers	4 512
	Tetramers	2 461
	Pentamers	733
	Hexamers	319
	Heptamers	114
	Octamers	59
	Nonamers	ND
	Polymers (DP>10)	1 038

ND: Not detected. DP: Degree of polymerization.

**Fig 1:**
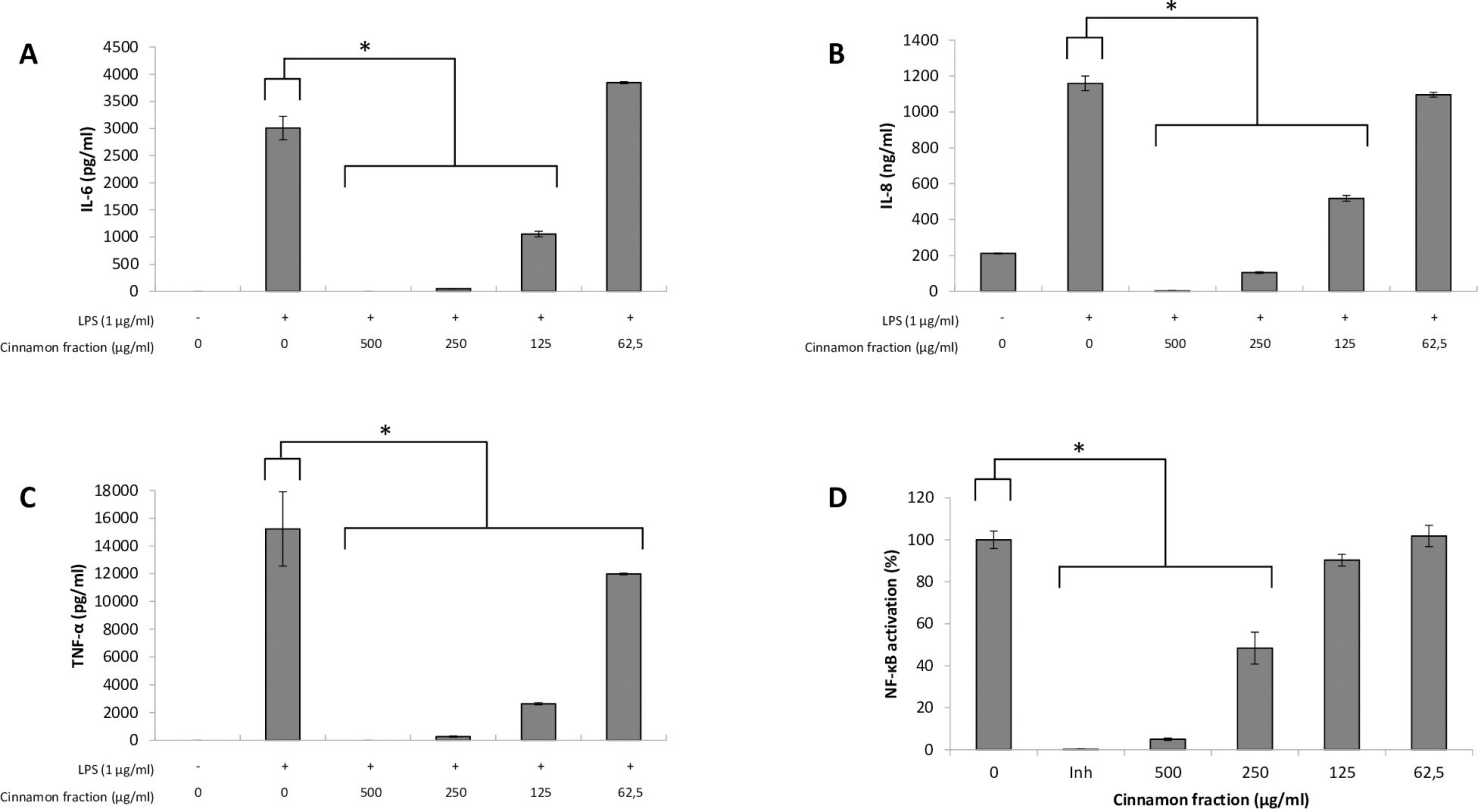
Effect of the cinnamon fraction on IL-6 (panel A), IL-8 (panel B), and TNF-α (panel C) secretion by macrophages and on NF-κB activation in monocytes stimulated with *A*. *actinomycetemcomitans* LPS. Assays were performed in triplicate in two independent experiments and the means ± SD were calculated. *, significant inhibition at *p* < 0.01.

**Fig 2:**
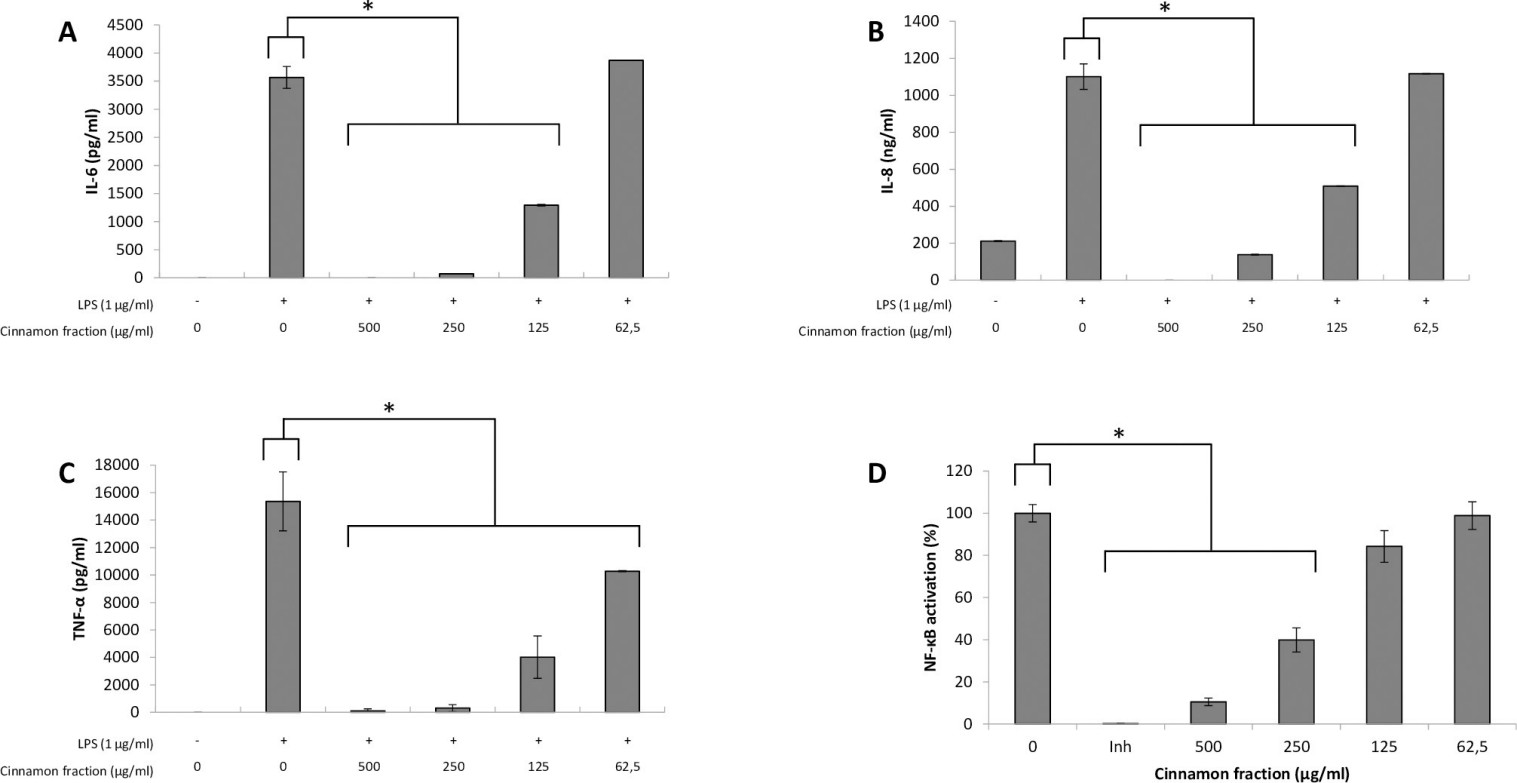
Effect of the cinnamon fraction on IL-6 (panel A), IL-8 (panel B), and TNF-α (panel C) secretion by macrophages and on NF-κB activation in monocytes stimulated with *E*. *coli* LPS. Assays were performed in triplicate in two independent experiments and the means ± SD were calculated. *, significant inhibition at *p* < 0.01.

We first investigated the effects of the cinnamon fraction on IL-6, IL-8, and TNF-α secretion in a macrophage model stimulated with LPS from either *A*. *actinomycetemcomitans* or *E*. *coli*. Adherent macrophages were pre-treated for 2 h with the cinnamon fraction and were then stimulated (24 h) with LPS (1 μg/mL). In the absence of the cinnamon fraction, the stimulation of the macrophages with either LPS significantly increased the secretion of the three cytokines (Figs 1 and 2). The two LPS induced cytokine secretion by macrophages in similar fashion, with TNF-α being secreted in the highest amounts. The increased cytokine secretion by LPS-stimulated macrophages was significantly and dose-dependently attenuated by the cinnamon fraction. More specifically, 250 μg/mL of the fraction reduced the secretion of IL-6, IL-8, and TNF-α by 98.3%, 91.0%, and 97.1%, respectively, by macrophages stimulated with *A*. *actinomycetemcomitans* LPS (Fig 1). The anti-inflammatory activity of the cinnamon fraction was confirmed by stimulating the macrophages with *E*. *coli* LPS. As shown in Fig 2, the cinnamon fraction significantly and dose-dependently inhibited cytokine secretion by *E*. *coli* LPS-stimulated macrophages. More specifically, 250 μg/mL of the fraction reduced the secretion of IL-6 by 97.9%, IL-8 by 87.5%, and TNF-α by 98.1% (Fig 2).

The ability of the cinnamon fraction to modulate the activation of the NF-κB transcription factor was determined using a subclone of the U937 cell line transfected with a luciferase gene coupled to a promoter with three NF-κB- binding sites. The fraction dose-dependently inhibited the activation of NF-κB induced by *A*. *actinomycetemcomitans* or *E*. *coli* LPS (Figs 1D and 2D). More specifically, 250 μg/mL of the cinnamon fraction reduced NF-κB activation by 51.6% and 60.1% with respect to stimulation with *A*. *actinomycetemcomitans* LPS and *E*. *coli* LPS, respectively. As expected, the commercial inhibitor BAY-11-7082 (25 μM), which was used as a positive inhibitory control, completely prevented NF-κB activation.

**Fig 3:**
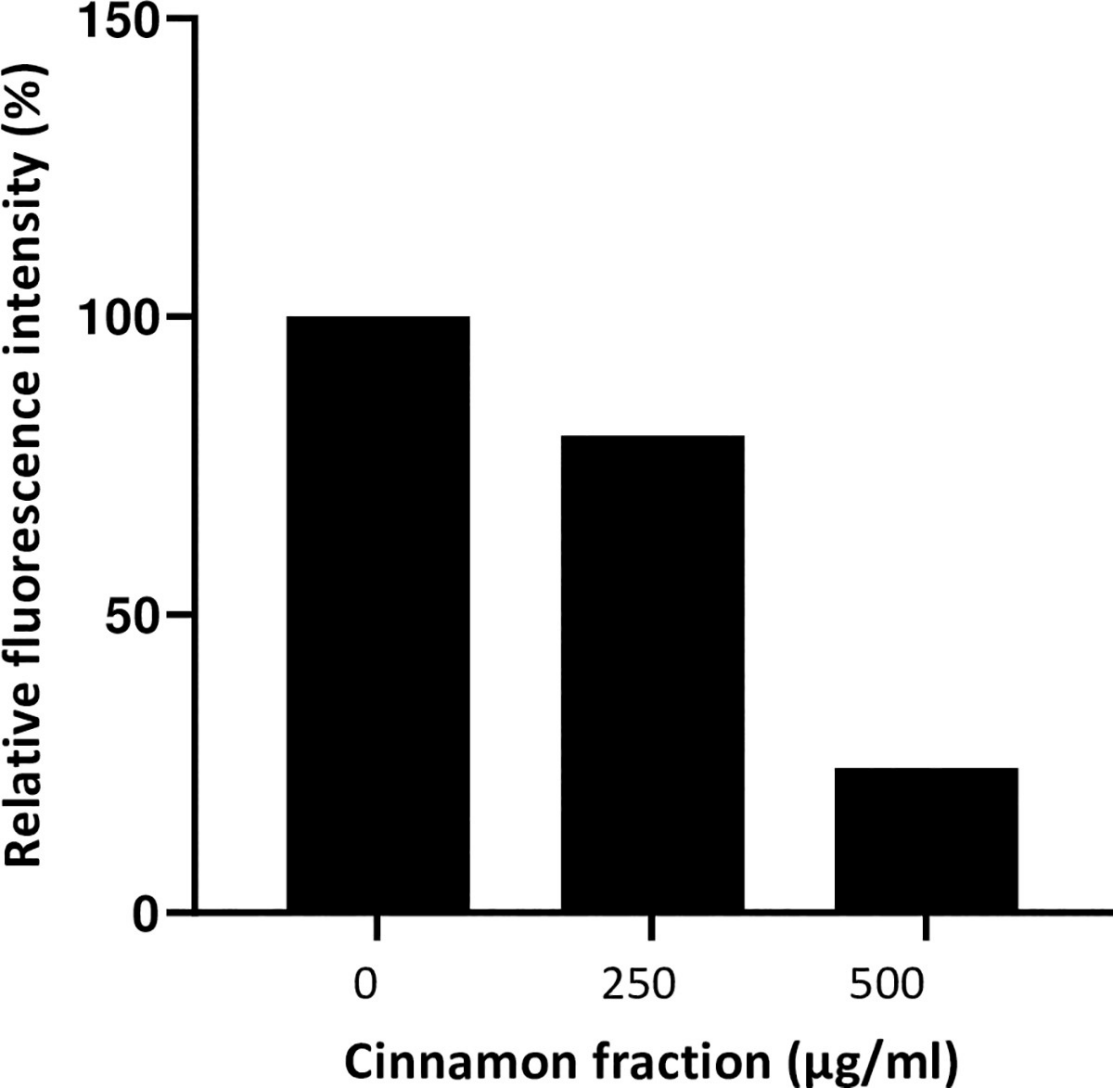
Effect of the cinnamon fraction on the binding of *E*. *coli* LPS-Alexa 488 to monocytes as determined by a flow cytometry analysis. Assays were performed in quadruplicate, and a representative set of data is presented.

We then investigated the effect of a pre-treatment of U937 cells with the cinnamon fraction on the subsequent binding of fluorescent *E*. *coli* LPS. As reported in Fig 3, 250 and 500 μg/mL of the cinnamon fraction inhibited the binding of LPS by 20.5% and 75.6%, respectively.

**Fig 4:**
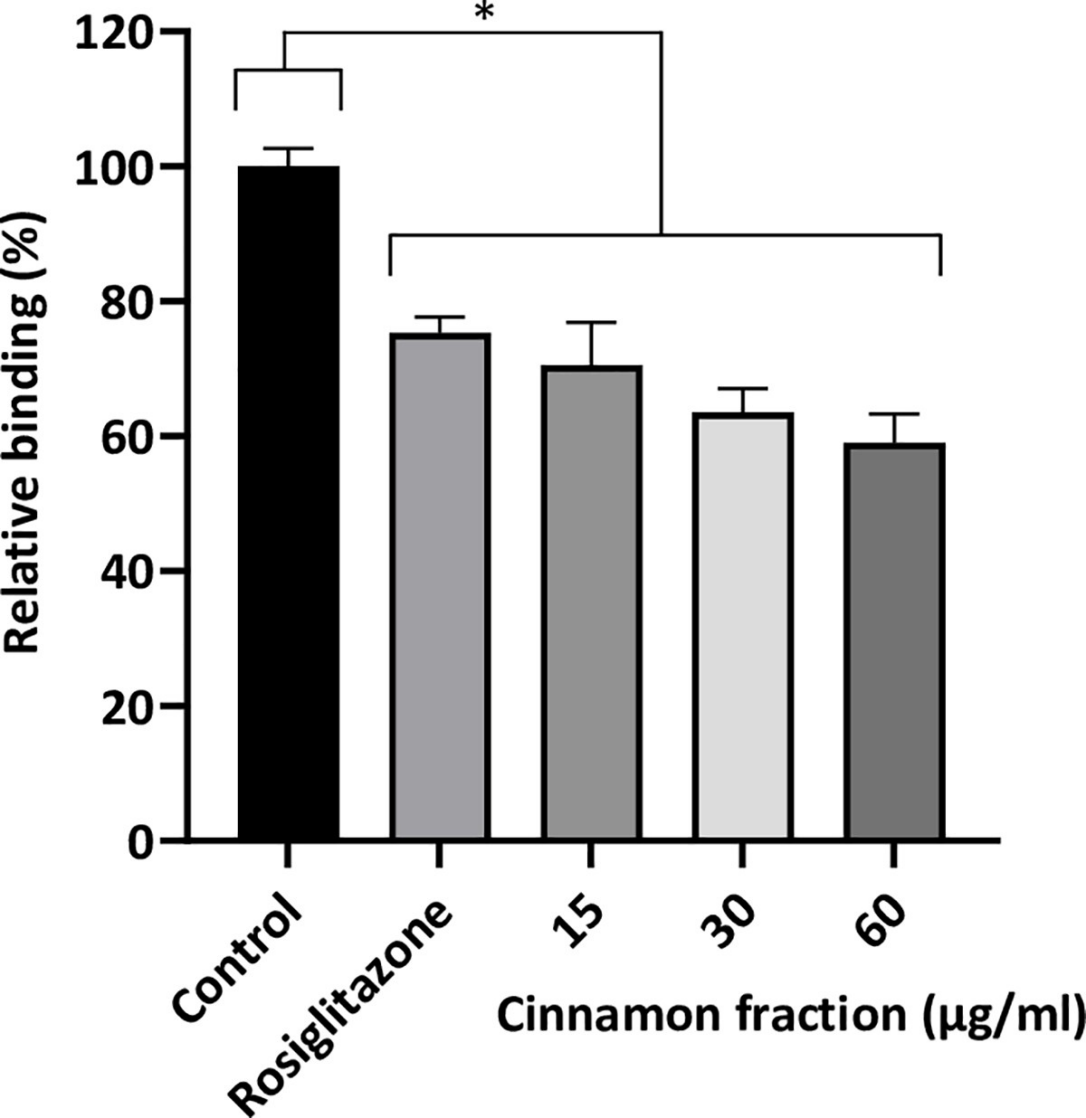
PPAR‐γ binding activity of the cinnamon fraction as determined using a PPAR‐γ competitor assay. Assays were performed in triplicate in two independent experiments and the means ± SD were calculated. *, significant inhibition at *p* < 0.01.

The binding of the cinnamon components to PPAR-γ was evaluated using a PPAR-γ competitor binding assay and was compared to rosiglitazone, a positive inhibitor control. As shown in Fig 4, the cinnamon fraction dose-dependently reduced binding between the fluorescent PPAR-γ ligand and PPAR-γ. The cinnamon fraction (60 μg/mL) inhibited binding by 40.8% compared to 24.6% with rosiglitazone.

## Discussion

Inflammation is a localized protective host response to injury or infection. An acute response, which is fast and short-lived, is associated with the elimination of the primary cause of the inflammation and the repair of the affected tissue. However, if the inflammation is not resolved, this results in a chronic state that plays a central role in numerous diseases, including rheumatoid arthritis, asthma, and periodontal disease. During periodontitis, the severe form of periodontal disease, inflammatory cells recruited in response to the bacterial challenge secrete a broad array of inflammatory mediators that mediate the destruction of both soft and hard tissue [6]. Given the key role played by the bacteria-mediated immuno-inflammatory process in the progression and severity of periodontitis, modulating this host inflammatory response may constitute a potential therapeutic strategy [11, 12]. Host modulation can be defined as a therapeutic approach aimed at restoring the balance between proinflammatory and anti-inflammatory mediators to stop the progression of the disease and to create an environment favorable to inflammation resolution and tissue repair [11].

We used a human macrophage model stimulated with *A*. *actinomycetemcomitans* or *E*. *coli* LPS to show that the cinnamon fraction dose-dependently reduces IL-6, IL-8, and TNF-α secretion with no cytotoxicity, regardless of the concentration used (data not shown). These three cytokines are thought to play a critical role in periodontitis given that their levels are elevated in the gingival crevicular fluid of chronic periodontitis patients and decrease following periodontal treatments [22, 23]. While IL-8 is a key chemokine that contributes to the recruitment of neutrophils and other leukocytes to sites of inflammation, IL-6 and TNF-α are major proinflammatory cytokines that play a role in osteoclastogenesis activation by upregulating the expression of the receptor of nuclear factor-kappa ligand (RANKL) and inhibiting osteoblast differentiation [24–26]. We recently showed that TNF-α also time-dependently damages epithelial keratinocyte tight junction barrier integrity, as shown by changes in transepithelial electrical resistance and FITC-dextran transport [27]. Given the above, the ability of the cinnamon fraction to attenuate the secretion of IL-6, IL-8, and TNF-α may thus promote a healthy periodontium.

The inflammatory response of immune cells to microbial challenges is mainly driven by the activation of the NF-κB signaling pathway [3]. It has been proposed that compounds with the ability to specifically prevent NF-κB activation may have a therapeutic potential for the treatment of inflammatory diseases, including periodontal diseases [28]. We used the U937-3xκ B-LUC cell line, which makes it possible to monitor NF-κB activation by recording luciferase activity, to show that the cinnamon fraction prevents LPS-induced NF-κB activation. This is in agreement with previous studies indicating that plant-derived phytochemicals show promise as potent and safe inhibitors of cancers and inflammatory disorders driven by NF-κB [29].

PPAR-γ, a ligand-dependent transcription factor of the nuclear receptor super family, can decrease the activation of NF-κB and thus attenuate the secretion of pro-inflammatory cytokines by immune cells such as macrophages [30, 31]. In recent years, PPAR-γ agonists have shown promise for the treatment of several inflammatory diseases [31–33]. We used a competitor assay to show that the cinnamon fraction may contain a natural PPAR-γ ligand.

LPS can bind CD14, a co-receptor located on the plasma membrane of immune cells, including macrophages, which subsequently activates toll-like receptor 4 (TLR-4) and triggers an intracellular signaling pathway that can induce the release of pro-inflammatory cytokines [34]. We showed that the cinnamon fraction can reduce the binding of LPS, which may be another mechanism that contributes to its anti-inflammatory property.

Several studies have reported that cinnamon constituents, mostly essential oils, exhibit anti-inflammatory activities [35–38]. Hong et al. [36] showed that the oral administration of a cinnamon (*Cinnamomum cassia*) bark water extract, whose detailed composition was not reported, in mice significantly decreases LPS-induced TNF-α levels in serum. More recently, Veilleux and Grenier [16] showed that the cinnamon fraction used in the present study attenuates the secretion of IL-6 and IL-8 by TNF-α-treated oral epithelial cells. The anti-inflammatory properties of the cinnamon fraction may be related to its high content in proanthocyanidins, which include both A-type and B-type linkages [39–41]. In previous studies, proanthocyanidins purified from blueberry and cranberry have been shown to reduce cytokine secretion and NF-κB activation in macrophages [42–44].

Recent studies have provided evidence suggesting that periodontal diseases may trigger a systemic inflammatory state leading to a number of other diseases or disorders, including rheumatoid arthritis, type 2 diabetes, cardiovascular diseases, and preterm low birth weight [10, 45]. The cinnamon fraction, which reduced the production of proinflammatory cytokines by LPS-treated macrophages, may thus have a positive impact not only for periodontal diseases but also for systemic inflammatory diseases.

Interestingly, a number of studies have reported that cinnamon constituents, mainly essential oils, possess antimicrobial activities against both Gram-positive and Gram-negative oral bacteria [15]. Current research in our laboratory is investigating the antibacterial and anti-biofilm properties of the cinnamon fraction against major periodontal pathogens, including *A*. *actinomycetemcomitans*. By exerting a dual action through effects on the two etiological components of periodontal disease, namely periodontal pathogens and the host inflammatory response, the therapeutic potential of the cinnamon fraction would be even greater.

Within the limitations of this study, such as the use of a single cell line in an *in vitro* model, the cinnamon fraction, commercialized as Cinnulin PF^®^, was shown to possess therapeutic potential for the treatment of periodontal diseases due to its anti-inflammatory properties. These observations should open the door for future clinical trials on the potential of this bioactive fraction to prevent and/or treat periodontal diseases. In this respect, the benefits provided by the use of oral-hygiene products (mouthrinses and chewing gums) or slow-release devices (inserted in diseased periodontal sites) containing the cinnamon extract merit being investigated.
